# Doppelbilder bei einem 60-jährigen Patienten

**DOI:** 10.1007/s00347-021-01421-5

**Published:** 2021-06-10

**Authors:** Amine Maamri, Shady Suffo, Barbara Käsmann-Kellner, Berthold Seitz

**Affiliations:** Klinik für Augenheilkunde, Universitätsklinikum des Saarlandes (UKS), Kirrberger Str. 100, Gebäude 22, 66421 Homburg/Saar, Deutschland

## Anamnese

Ein 60-jähriger Patient stellte sich in unserer Sprechstunde wegen binokularer horizontaler Doppelbilder beim Blick nach rechts vor. Der Patient ist in Deutschland und nicht äquatornah aufgewachsen. In der Augenanamnese wurden bei unserem Patienten beim Rezidiv eines Pterygiums am rechten Auge 4‑mal Pterygiumexzisionen mit freiem ipsilateralem und kaudotemporalem Bindehauttransplantat und Mitomycin C (MMC) im Jahr 2018 durchgeführt. Im Jahr 2019 erfolgte bei erneutem Rezidiv des Pterygiums eine fünfte Pterygiumexzision mit MMC und Amnionmembrantransplantation (AMT) sowie einem freien ipsilateralen Bindehauttransplantat mit Limbustransplantation in unserer Klinik. Außerdem gab der Patient bei der familiären Anamnese an, dass seine älteste Tochter auch ein Pterygium habe.

## Klinischer Befund

Am Vorstellungstag im Jahr 2020 betrug der bestkorrigierte Visus 0,9 rechts und 1,25 links. Der Augeninnendruck betrug applanatorisch 14 mm Hg an beiden Augen.

**Figure 1:**
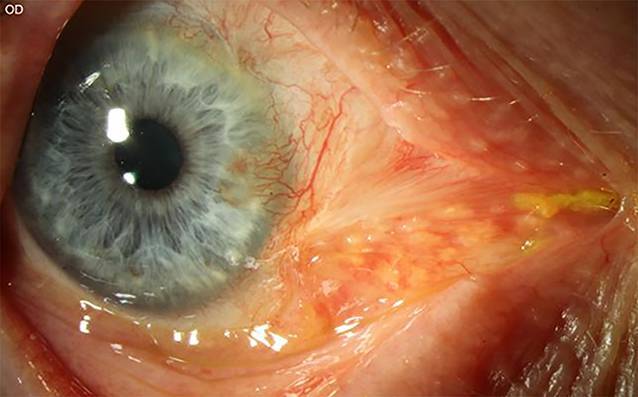

Spaltlampenmikroskopisch zeigte sich am betroffenen rechten Auge ein Pterygiumrezidiv nasal mit einer Basis von 5 mm und einer Höhe von 2,5 mm. Eine Stocker-Linie war nicht sichtbar. Außerdem zeigten sich ein Symblepharon von dem medialen Oberlid bis nach 4 Uhr paralimbal sowie eine Plica-Dislokation nach zentral (Abb. 1). Fundoskopisch zeigten sich am rechten Auge keine Auffälligkeiten.

## Orthoptischer Status

In der Primärposition zeigte sich ein Parallelstand der Augen ohne Einstellbewegung.

**Figure 2:**
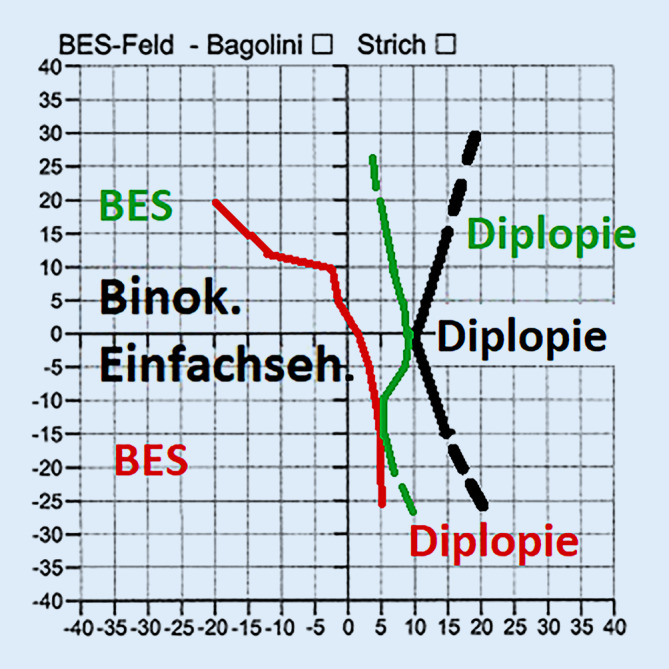

Eine Diplopie fand sich bereits bei 2,5° Rechtsblick und führte zu einer kompensatorischen Kopfzwangshaltung (Abb. 2). Die Diplopie belastete den Patienten subjektiv sehr stark im täglichen Leben.

## Diagnostik

### Pentacam (Oculus, Wetzlar, Deutschland)

In der Pentacam zeigte sich präoperativ am rechten Auge topographisch ein irregulärer Astigmatismus mit kornealem Astigmatismus von 0,9 dpt. Tomographisch zeigte sich am rechten Auge eine nasale Verdickung von 624 µm. Bei der letzten Kontrolle zeigte sich am rechten Auge topographisch ein irregulärer Astigmatismus mit kornealem Astigmatismus von 1,3 dpt. Tomographisch zeigte sich am rechten Auge keine nasale Verdickung mehr.

## Wie lautet Ihre Diagnose?

## Definition

Das Pterygium ist eine multifaktoriell induzierte degenerative Erkrankung der limbalen Bindehaut. Es handelt sich histologisch um eine fokale fibrovaskuläre Proliferation der Bindehaut auf die Hornhaut im Sinne einer fokalen Limbusstammzelleninsuffizienz. Die Risikofaktoren einer Pterygiumentwicklung sind eine chronische Exposition mit ultraviolettem (UV) Licht neben hereditären Faktoren, proinflammatorische und angiogene Zytokine sowie eine veränderte Remodellierung der extrazellulären Matrix [1, 2]. Die Symptome eines Pterygiums sind Oberflächenprobleme (entzündlich und durch Tränenfilmfehlverteilung) und ästhetische Beschwerden. Beim fortgeschrittenen Pterygium ist eine Visusminderung durch Induktion eines irregulären Astigmatismus zu erwarten, ohne dass das Pterygium die Pupillarzone erreicht. In der klinischen Studie von Seitz et al. schien der Visus bis zu einem Schwellenwert von 2,5 mm für die Höhe des Pterygiums weitgehend unbeeinflusst zu sein [8]. Bei schwerem Rezidiv eines Pterygiums mit ausgeprägter Narbenbildung können darüber hinaus Doppelbilder aufgrund einer eingeschränkten Bulbusmotilität mit Kopfzwangshaltung – wie bei unserem Patienten – beobachtet werden [1].

## Therapie und Verlauf

Indikationen zur Operation eines Pterygiums sind Visusminderung, Zunahme des Astigmatismus, drohende Invasion in der optischen Achse, sekundäre Diplopie sowie Oberflächenbeschwerden [1]. Die Pterygiumexzision mit freiem Bindehauttransplantat ist die Methode der ersten Wahl, auch bei rezidivierendem bilateralem doppelköpfigem (temporalem und nasalem) Pterygium [4].

Wir haben bei unserem Patienten eine Pterygiumexzision mit freiem Bindehauttransplantat, Symblepharolyse, 3 min Applikation von MMC 0,02 mg/ml und eine episklerale AMT als Graft End-zu-End mit der ortsständigen und transplantierten Bindehaut in Intubationsnarkose mit Einsetzen einer Illig-Schale durchgeführt. Hierbei wurde intraoperativ der M. rectus medialis angeschlungen, um ihn bei der Präparation des extensiven Narbengewebes nicht zu durchtrennen.

**Figure 3:**
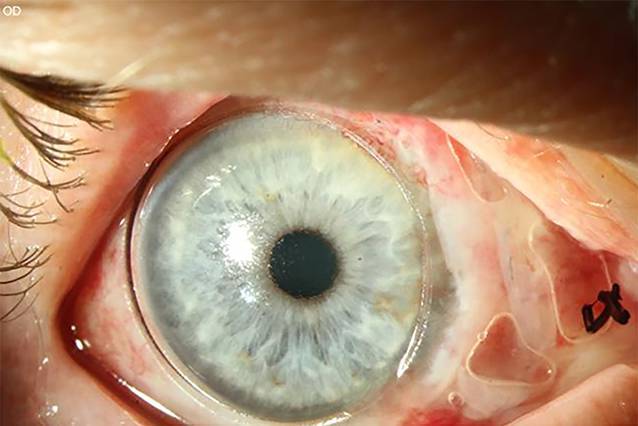

Am ersten postoperativen Tag zeigte sich am operierten rechten Auge eine adaptierte Bindehaut. Das Transplantat und die Illig-Schale zur Symblepharonprophylaxe waren in loco. Es zeigte sich eine nasale Hornhauterosion (Abb. 3).

Im orthoptischen Status zeigte sich in der Primärposition ein Parallelstand der Augen ohne Einstellbewegung. Diplopie bestand ab 10° Rechtsblick (Abb. 2). Postoperativ erhielt der Patient am rechten Auge als lokale Therapie Hyaluronsäure-AT 5‑mal/Tag, dexa- und gentamycinhaltige Augensalbe (AS) 5‑mal/Tag, Illig-Schale, MMC 0,02 % 4‑mal/Tag und Ciclosporin 0,1 % AT zur Nacht. Mitomycin C ist ein Antibiotikum und ein Antikarzinogen, das aus *Streptomyces caespitosus* extrahiert wird und die Proliferation von Fibroblasten hemmt. Mitomycin C wirkt auch auf normales Gewebe. Daher sind Komplikationen wie Skleranekrosen, Hornhautperforation, Hornhautödem, Sekundärglaukom, Hornhautverkalkung und Katarakt möglich. Daher muss Mitomycin C vorsichtig angewendet werden. Im Gegensatz dazu führt die Cyclosporin-Augentropfen-Anwendung nach Pterygiumexzision nur zu geringen Komplikationen wie Irritation, Brennen und Hyperämie [5]. Systemisch erhielt er intraoperativ Solu Decortin H (SDH) 150 mg intravenös und ab dem ersten postoperativen Tag Steroidtabletten 100 mg, die alle 2 Tage um 20 mg reduziert wurden mit Magenschutz während der Kortisontherapie. Dies galt auch als Standardtherapie nach Pterygiumexzision zur Vermeidung postoperativer Rezidive an der Universitätsaugenklinik Erlangen [1].

**Figure 4:**
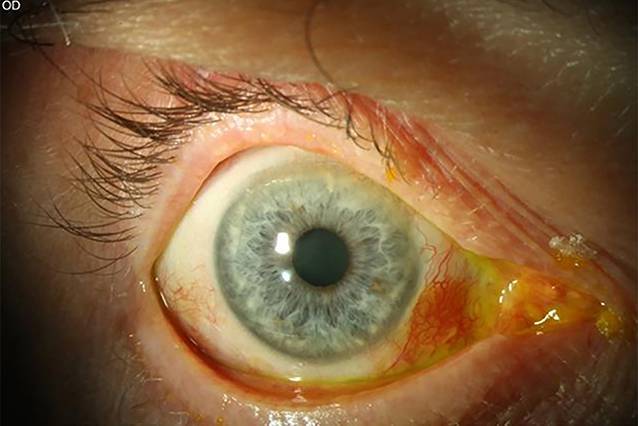

Bei der letzten postoperativen Kontrolle 6 Monate postoperativ zeigte sich ein reizfreier Befund. In der Primärposition hatte der Patient nach wie vor keine Doppelbilder. Der bestkorrigierte Visus betrug am rechten Auge 0,8, und der Druck lag bei 12 mm Hg. Spaltlampenbiomikroskopisch zeigte sich kein Pterygiumrezidiv mehr (Abb. 4). Fundoskopisch waren die Befunde regelrecht. Im orthoptischen Status lag Diplopie ab 10° Rechtsblick vor (Abb. 2). Das Feld binokularen Einfachsehens war bereits am ersten postoperativen Tag auf 10° Rechtsblick erweitert. Das bedeutete eine große subjektive Verbesserung für den Patienten. Diese subjektive und objektive Verbesserung war auch nach 6 Monaten noch erhalten. Postoperativ nahm der Patient keine Kopfzwangshaltung mehr ein, somit war ein Prismenausgleich nicht mehr erforderlich.

Der Pathomechanismus der Rezidive des Pterygiums ist multifaktoriell. Wichtige Faktoren scheinen eine chronische UV-Exposition, hereditäre Faktoren sowie proinflammatorische und angiogene Zytokine zu sein. Die UV-Exposition hat mehrere Folgen, die die Entstehung einer fibrovaskulären Proliferation begünstigen. Zum einen wird spekuliert, dass das UV-Licht eine Destruktion der limbalen Stammzellen verursacht und es dadurch zu einem Verlust der limbalen Barrierefunktion kommt. Des Weiteren scheint das UV-Licht zu einem verstärkt proinflammatorischen und angiogenen Milieu zu führen und damit zur Angiogenese und Proliferation. Das UV-Licht führt außerdem zu einer Inaktivierung von Tumorsuppressorgenen, die eine unkontrollierte Proliferation von vaskulären Zellen und Fibroblasten begünstigt.

Als Rezidivprophylaxe spielen Mitomycin-C-Tropfen intraoperativ zur Hemmung der Fibroblastenproliferation eine wichtige Rolle. Die konsequente UV-Vermeidung mit stark UV blockierenden Brillengläsern und Sonnenbrillen erwies sich als wichtigster postoperativer Schutz gegen ein Rezidiv [1].

**Diagnose:** Mechanische Abduktionsblockade im Rahmen eines sechsten Pterygiumrezidivs mit schwerem Symblepharon

Die Ergebnisse einer 4‑Patienten-Studie aus Erlangen legt nahe, dass die Kombination aus ipsilateraler autologer Limbustransplantation und homologer Amnionmembrantransplantation effektiv zur Behandlung eines rezidivierenden Pterygiums mit Symblepharonbildung eingesetzt werden kann [6]. Eine andere retrospektive Studie von Jordan et al. unterstreicht die Sicherheit und Wirksamkeit des konjunktivalen Limbusautotransplantats zur Verhinderung des Wiederauftretens von rezidivierendem Pterygium [7].

Leider sind Rezidive beim Pterygium häufig und können mit massiver subkonjunktivaler Vernarbung, Symblepharonbildung und Diplopie einhergehen [2, 3]. Allerdings wurde ein sechstes Pterygiumrezidiv – wie in unserem Fall – in der Literatur noch nicht beschrieben. Als Limitation unseres Falls muss das grenzwertig kurze Follow-up erwähnt werden. In der Literatur [5] wird eine Nachbeobachtungszeit von mindestens 1 Jahr empfohlen. Daher haben wir mit unserem Patienten einen erneuten Termin 1 Jahr nach der Operation vereinbart – danach jährlich.

## Fazit für die Praxis

- Die Indikationen zur Operation eines Pterygiums sind Visusminderung, Zunahme des Astigmatismus, drohende Invasion in die optische Achse, sekundäre Diplopie sowie Oberflächenbeschwerden.
- Um ein Rezidiv nach Pterygiumexzision zu minimieren, kommen einige Maßnahmen in Betracht wie eine UV-Protektion, eine freie autologe Bindehauttransplantation von temporal unten, die intraoperative MMC-Applikation und die postoperative lokale Therapie mit Steroiden.
- Auch beim sechsten Pterygiumrezidiv mit Symblepharonbildung und Dislokation der Plica kann eine erneute Exzision von Pterygium und subkonjunktivalem Narbengewebe mit AMT und MMC-Applikation ein weiteres Rezidiv vermeiden helfen.
- Das Feld binokularen Einfachsehens kann durch die Motilitätsverbesserung nach Narbenlösung vergrößert werden.
